# Response Requirements Shape the Spatial Coding of Location-Specific Adjustments to Conflict Frequency

**DOI:** 10.1162/OPMI.a.340

**Published:** 2026-03-15

**Authors:** Peter Wühr, Herbert Heuer

**Affiliations:** TU Dortmund University, Dortmund, Germany; Leibniz Research Center for Working Environment and Human Factors (IfADo), Dortmund, Germany

**Keywords:** Simon task, conflict-frequency, proportion congruent, transfer, dimension weighting

## Abstract

Humans adjust to the frequency of conflicting stimuli so that the detrimental behavioral effects of frequent conflicts become smaller than those of rare conflicts. These adjustments become contingent on the locations where frequent and infrequent conflicts have been encountered. Experimentally such phenomena are studied by means of conflict tasks, a prominent one being the Simon task of the present experiments. Our Simon task involved four stimulus locations (upper-left, upper-right, lower-left, lower-right). Conflict frequency was manipulated for two diagonally opposite locations. For example, conflict frequency was low at the upper-left location and high at the lower-right location. At the remaining (non-manipulated) locations conflict frequency was intermediate. In Experiment 1 participants responded to stimulus colors by pressing a left or right key, in Experiment 2 by pressing a lower or upper key. We observed larger conflict effects for the low-conflict location than for the high-conflict location. Crucially, these adjustments to conflict frequency transferred to non-manipulated locations depending on the response configuration: transfer regions were the left or right hemifield with left-right responses, but the lower or upper hemifield with lower-upper responses. Assuming that transfer regions around manipulated stimulus locations are defined by identical (or similar) spatial codes, the pattern of transfer suggests a process of weighted two-dimensional location coding. According to this notion, the spatial dimension that is relevant for response discrimination has stronger weight in the coding of stimulus locations than a response-irrelevant dimension, and can therefore produce anisotropic transfer of conflict-frequency effects around manipulated locations.

## INTRODUCTION

Different stimuli, and even different features of the same stimulus, can activate different responses that compete for selection. Such response conflicts can be resolved by increasing attention to relevant stimulation and/or by decreasing attention to irrelevant stimulation. For example, when driving through a town, traffic lights are relevant stimuli whereas neon lights of shops and advertisements are irrelevant stimuli. A busy street with many shops and advertisements is an environment where distraction and response conflicts occur frequently, and therefore it imposes high demands on our ability to ignore distraction and to prevent or overcome response conflicts. In contrast, a calm street without shops and advertisements is an environment where distraction and response conflicts rarely occur, and therefore it imposes low demands on our ability to ignore distraction and to overcome response conflicts. Here we show, first, the learning of associations between different locations (such as streets) and different frequencies of response conflict and the adaption to them in a location-specific manner. Second, we explore to which other locations location-specific adaptation to conflict frequency transfers and how this transfer depends on response requirements. In the following we develop the specific issues addressed in a stepwise manner, beginning with a brief description of experimental conflict tasks.

### Experimental Conflict Tasks

In experimental conflict tasks, researchers vary the match (congruent condition) or mismatch (incongruent condition) between irrelevant stimulus and relevant stimulus and/or response features. The most prominent conflict tasks are the Stroop task, the Simon task, and the flanker task. In a typical Stroop task, participants name the ink color (relevant stimulus feature: color) of congruently or incongruently colored color words (irrelevant stimulus feature: word meaning). Faster color-naming responses to congruent than to incongruent stimuli demonstrate that participants cannot ignore the word meaning which creates a response conflict in incongruent conditions (e.g., Stroop, 1935; see MacLeod & MacDonald, 2000, for reviews). In a typical Simon task, participants press a left or right key in response to the color (relevant feature) of a stimulus that randomly appears at a left or right location (irrelevant feature). Faster responses in spatially congruent than in incongruent conditions again demonstrate that participants cannot ignore the irrelevant stimulus feature, that is, the location of the stimulus (e.g., Simon & Rudell, 1967; Wallace, 1971; see Hommel, 2011, and Lu & Proctor, 1995, for reviews).

Whereas response conflicts in Stroop and Simon tasks are caused by highly overlearned, possibly hard-wired, associations between irrelevant stimulus features and responses (e.g., Kornblum et al., 1990; MacLeod & MacDonald, 2000), response conflicts in the flanker task are caused by task instructions which map both relevant and irrelevant features to responses (e.g., Eriksen & Eriksen, 1974; Eriksen & Schultz, 1979; Fournier & Eriksen, 1990). In a typical flanker task, strings of letters are presented and participants are instructed to respond only to the central target letter (relevant feature) and to ignore the adjacent flankers (irrelevant feature). For example, target letters A and B may call for a right keypress and C and D for a left keypress. Responses are faster in congruent conditions, where the target and the flankers call for the same response (e.g., AAAAA, AABAA) than in incongruent conditions, where the target and the flankers call for different responses (e.g., AACAA, DDBDD; Eriksen & Eriksen, 1974; Grice et al., 1982; see Eriksen, 1995, for a review).

The Stroop, Simon, and flanker effects, that is, the differences between incongruent and congruent conditions in these conflict tasks, are generally attributed to activation or priming of responses by the irrelevant stimulus features which facilitates correct responses in congruent and inhibits them in incongruent conditions (e.g., Kornblum et al., 1990; Zhang et al., 1999). Although the different conflict tasks share this influence of the irrelevant stimulus feature (e.g., Ulrich et al., 2015), more detailed analyses of the differences between the response-time distributions in congruent and incongruent conditions reveal that they are not fully equivalent. In particular, conflict tasks differ in their dynamics, that is, in how congruency effects increase and/or decrease across the range of reaction times (e.g., Mackenzie et al., 2022; Mittelstädt et al., 2022). Thus, findings obtained with one type of conflict task cannot always be generalized to other conflict tasks.

### Location-Specific Effects of Conflict Frequency

The size of congruency effects depends on the relative frequencies of congruent and incongruent trials. For example, the Stroop effect is larger in blocks with many congruent (and few incongruent) trials than in blocks with many incongruent (and few congruent) trials (e.g., Lindsay & Jacoby, 1994; Logan, 1980; Logan & Zbrodoff, 1979; Lowe & Mitterer, 1982), called list-wise ‘proportion congruent’ (PC) effect. PC effects have also been reported for the Simon task (e.g., Hommel, 1994; Stürmer et al., 2002; Wühr et al., 2015) and the flanker task (e.g., Gratton et al., 1992; Wendt & Luna-Rodriguez, 2009).

Modulations of congruency effects do not only arise when conflict frequency varies between blocks of trials, but also when it varies between stimulus locations within the same block of trials (e.g., Corballis & Gratton, 2003; Crump et al., 2006; Wendt et al., 2008). In a seminal study of location-specific PC effects, Corballis and Gratton (2003, Experiment 2) used a flanker task with two different stimulus positions: a target and four congruent or incongruent flankers were presented either to the left or to the right of the center of a computer screen. For each participant, the stimuli were mostly congruent (i.e., 75% congruent vs. 25% incongruent) at one location, but mostly incongruent (i.e., 25% congruent vs. 75% incongruent) at the other location. Hence, across both locations, congruent and incongruent trials were equally frequent. Corballis and Gratton (2003) observed a larger congruency effect at the location with mostly congruent stimuli than at the location with mostly incongruent stimuli. Location-specific PC effects have not only been observed with the flanker task (e.g., Colvett et al., 2023; Corballis & Gratton, 2003; Weidler et al., 2020), but also with the Stroop task (e.g., Crump et al., 2006; Crump & Milliken, 2009), and with the Simon task (Hübner & Mishra, 2016).

There are two broad accounts of PC effects (see Bugg & Crump, 2012, for review). According to *control-based* accounts, the modulations of congruency effects are the result of flexible cognitive control (e.g., Gratton et al., 1992; Lindsay & Jacoby, 1994; Logan, 1980; Lowe & Mitterer, 1982; Wendt & Luna-Rodriguez, 2009). The most prominent control-based account, conflict-monitoring theory (e.g., Botvinick et al., 2001; cf. Ullsperger et al., 2014), holds that response conflicts are detected and attentional weights for relevant and/or irrelevant stimulus features are adjusted accordingly. A relative increase of attention towards the relevant stimulus in the case of many incongruent trials can be achieved by increasing attention towards the relevant stimulus and/or decreasing attention towards the irrelevant stimulus. To account for location-specific PC effects, control-based accounts assume that humans can adopt different attentional control settings for different locations in the visual field and flexibly switch between them (e.g., Corballis & Gratton, 2003; Crump et al., 2006; Crump & Milliken, 2009; Weidler et al., 2020).

According to *learning-based* accounts, the modulations of congruency effects are the result of learning stimulus-response contingencies (e.g., Melara & Algom, 2003; Schmidt, 2013; Schmidt & Besner, 2008; Schmidt et al., 2007). In a condition with many congruent trials, the relevant stimulus feature—and thus the correct response—occurs more often together with a congruent than with an incongruent irrelevant stimulus feature, and therefore the congruent irrelevant feature becomes associated with the correct response. In contrast, in a condition with many incongruent trials, the relevant stimulus feature occurs more often together with an incongruent than with a congruent irrelevant feature so that the incongruent irrelevant feature becomes associated with the correct response. In the case of many incongruent trials, the learned contingencies would serve to speed up correct responses in incongruent trials and slow them down in congruent trials, thereby reducing congruency effects; in the case of many congruent trials the learned contingencies would produce the opposite effects. To account for location-specific PC effects, learning-based accounts posit that different contingencies between irrelevant stimulus features and correct responses can be learned for different (stimulus) locations in the visual field (e.g., Schmidt, 2016, 2021).

Control-based and learning-based accounts of the modulation of congruency effects can be distinguished by the criterion of *item-specificity*. Whereas the effects of learned associations should be specific for the irrelevant stimuli actually used during learning, the effects of control-adjustments should be global and thus generalize to other stimuli. In tests of item-specificity, the proportion of congruent trials is manipulated for one subset of stimuli, called inducer items, whereas for another subset of stimuli, called the diagnostic items, the frequencies of congruent and incongruent trials are held constant (see Braem et al., 2019, for a description of the method). Transfer from inducer items to diagnostic items has been observed both for list-wise PC effects (e.g., Bugg & Chanani, 2011) and location-specific PC effects (e.g., Crump et al., 2017; Crump & Milliken, 2009; Weidler et al., 2022), providing evidence for a role of cognitive control in both types of PC effects.

### Spatial Transfer of Location-Specific Effects of Conflict Frequency

Location-specific PC effects can transfer from frequency-manipulated stimulus locations, that is, from those locations where conflict frequency is high and low, respectively, to non-manipulated locations, where congruent and incongruent trials have equal frequencies (e.g., Corballis & Gratton, 2003; Pickel et al., 2019; Weidler & Bugg, 2016). In their Experiment 3, Corballis and Gratton (2003) used a flanker task with letter strings of width 6° of visual angle. These stimuli were presented randomly at three different locations on the computer screen: at 4° to the left of screen center, at screen center, or at 4° to the right of screen center. In each block of trials the proportion of congruent trials was high or low at both peripheral locations (manipulated locations), whereas at the central (non-manipulated) location congruent and incongruent trials had equal frequency. The authors observed a list-wise PC effect for the peripheral locations: congruency effects were larger for mostly congruent than for mostly incongruent stimuli. Crucially, this list-wise PC effect transferred from the peripheral to the central location where the congruency effect was larger when mostly congruent stimuli occurred at the peripheral locations than when mostly incongruent stimuli occurred at the peripheral locations.

To account both for location-specific PC effects (as observed in their Experiment 2, described above) and for the transfer of list-wise PC effects from the peripheral locations to the central location, Corballis and Gratton (2003) proposed separate conflict-adaptation mechanisms in the two cortical hemispheres, each one producing adjustment to conflict arising from stimuli in the contra-lateral visual hemifield. When conflict frequency differs between peripheral locations, as in their Experiment 2, the two hemispheres adjust independently; when conflict frequencies are the same at the peripheral locations, as in Experiment 3, adaptation transfers to the central location because this location belongs to both hemifields.

Subsequent studies replicated spatial transfer of location-specific PC effects with different types of conflict task, mostly variants of the flanker task (e.g., Kuratomi & Yoshizaki, 2013; Weidler & Bugg, 2016; Weidler et al., 2020; but see Wendt et al., 2008, for negative results). Pickel et al. (2019) observed transfer of PC effects from manipulated to non-manipulated locations in a spatial Stroop task, but not in a classic Stroop task with conflict between colors and color words. From this result they concluded that “spatial information must be a source of conflict to facilitate learning of spatial categories within which control settings transfer” (Pickel et al., 2019, p. 2795). Although spatial information is clearly a source of conflict in the Simon task, transfer of location-specific PC effects in the Simon task has not been investigated so far.

### Regions of Spatial Transfer of Location-Specific PC Effects

Location-specific PC effects transfer to other locations, but the very existence of location-specific PC effects indicates that spatial transfer must be limited. Otherwise the effects of different proportions of incongruent trials at different locations should (partially) cancel each other out, so that location-specific PC effects would be absent (or only small). Thus, it remains to specify the boundaries of spatial transfer, that is, the regions within which spatial transfer can be observed, “transfer regions” for short.

For example, if there were separate mechanisms for the left and right visual hemifields, transfer should occur to new positions within the same lateral hemifield, but not to new positions in the contralateral hemifield. As far as spatial transfer is concerned, manipulated and non-manipulated locations within transfer regions would be coded identically, for example both as “left” or both as “right” depending on the hemifield. In contrast, non-manipulated locations outside the transfer region of the manipulated location would receive a different code. An obvious alternative to such categorical coding would be coding in terms of spatial continua. Transfer would then be expected primarily for non-manipulated locations that are sufficiently close to manipulated ones.

For stimuli in conflict tasks categorical spatial coding in terms of, e.g., left and right rather than continuous coding in terms of spatial distances has been suggested by Weidler et al. (2020). This suggestion was based on flanker-task studies with four stimulus locations, one location with mostly congruent stimuli, one location with mostly incongruent stimuli (manipulated locations), and two locations (non-manipulated locations) with equal frequencies of congruent and incongruent stimuli. Each of the non-manipulated locations was situated closer to one of the manipulated locations. With this setup, transfer was not only observed when the non-manipulated locations were located between the manipulated ones (e.g., Corballis & Gratton, 2003; Pickel et al., 2019), but also when they were situated more peripherally than the manipulated locations (e.g., Weidler et al., 2020; see also Kuratomi & Yoshizaki, 2013).

Weidler et al. (2022) showed that location-specific PC effects do not require locations in the left and right visual hemifields, but can also be observed when they are above and below fixation (see also, Colvett et al., 2023). Of course, the finding of different PC effects at different locations implies that these locations are coded differently, so that each of the different PC effects is not reduced or even abolished by transfer from the alternative PC effect. Different location-specific PC effects can even be observed when the two locations are in the same hemifield at different lateral eccentricities, and these location-specific PC effects transferred to non-manipulated locations above the manipulated ones. Hence, the categorical coding of stimulus locations is not restricted to the right and left visual hemifields and not even to the horizontal dimension. Weidler et al. (2022, p. 321) concluded that “participants are employing categorical encoding even when both locations appear in the same coarse category of space (for example, by coding the two locations as ‘left’ and ‘further left’). However, instead of a categorical coding as “left” and “further left” relative to the body midline (or the fixation point on the computer screen), there can be alternative frames of reference for categorical coding as “left” and “right” (cf. Umiltà & Liotti, 1987; Wang et al., 2016). For example, two stimulus positions can be coded as “left” and “right” (or “below” and “above”) relative to each other, no matter where in the field of view they are located.

Weidler and colleagues attributed spatial transfer of location-specific PC effects to categorical spatial coding, but spatial proximity between manipulated and non-manipulated locations may have contributed to their results. In their experiments, one non-manipulated location was closer to the mostly congruent location, and the other non-manipulated location was closer to the mostly incongruent location (Pickel et al., 2019; Weidler & Bugg, 2016; Weidler et al., 2020, 2022). Results showed that performance at a non-manipulated location was more strongly affected by the PC manipulation at the closer manipulated location than by the PC manipulation at the more distant manipulated location. Hence, transfer from manipulated locations could have resulted from categorical coding and/or from spatial proximity (evidence for an effect of spatial proximity on location-specific PC effects is described in Diede & Bugg, 2016, 2019).

### The Present Study

The present experiments have two aims. The first one is to consolidate the evidence of location-specific effects of conflict frequency in the Simon task. We are aware of only one previous study of location-specific PC effects in this task by Hübner and Mishra (2016) which reported rather small effects. Our variant of the Simon task used a square configuration of stimulus locations (upper-left, upper-right, lower-left, lower-right) and manipulated conflict frequency for two diagonally opposite locations (e.g., 83% congruent and 17% incongruent stimuli at the upper-left location versus 17% congruent and 83% incongruent stimuli at the lower-right location). We expected location-specific PC effects: the Simon effect should be larger for the mostly congruent location than for the mostly incongruent location, corroborating the findings of Hübner and Mishra (2016).

The second and more important aim was to explore the coding of stimulus locations which governs the transfer of conflict-frequency effects from manipulated to non-manipulated locations. As the non-manipulated stimulus locations were both equidistant from the two manipulated ones, we asked (a) whether effects of conflict frequency would transfer from manipulated to non-manipulated locations at all or cancel each other out, and (b), if spatial transfer occurred at all, what would be the transfer region. If transfer is dominated by spatial proximity between manipulated and non-manipulated locations, we should not see a difference between the two non-manipulated locations. Different predictions arise, however, if transfer is not dominated by spatial proximity, but by the similarity between the spatial codes assigned to manipulated and non-manipulated locations. According to the categorical-coding hypothesis, participants might classify the stimulus locations in our task in four categories (“upper-left”, “upper-right”, “lower-right”, “lower-left”), so that there would be no spatial transfer at all. Categorical codes could also be “left” and “right”, possibly related to the lateral visual hemifields. According to this hypothesis, transfer should occur to non-manipulated locations in the same lateral hemifields as the manipulated locations (see also Corballis & Gratton, 2003). Finally, categorical codes could be “upper” and “lower”, so that transfer would be to non-manipulated locations in the same vertical hemifields as the manipulated ones.

Our stimulus configuration is ambiguous regarding lateral versus vertical categorical coding of the manipulated stimulus locations, but lateral or vertical coding could be induced by non-visual factors. Hemisphere-specific adaptations to conflict frequency would be such a factor, but this notion is at variance with recent findings (e.g., Weidler et al., 2022). We envisaged another potentially disambiguating factor, namely the spatial dimension on which responses differ, and varied it in two experiments: response locations differed laterally (left vs. right) in Experiment 1 and vertically (upper vs. lower) in Experiment 2. If the coding of stimulus locations were affected by the coding of responses, we should find left-right codes in Experiment 1 with spatial transfer within lateral hemifields, but upper-lower codes in Experiment 2 with spatial transfer within vertical hemifields. This would be another instance of the disambiguation of ambiguous visual stimuli by actions (e.g., Mitsumatsu, 2009; Veto et al., 2018; Wohlschläger, 2000), specifically how an ambiguous lateral versus vertical categorization of stimulus locations is disambiguated by the lateral versus vertical arrangement of response locations. From a broader perspective this would be another instance of common representations of visual and motor features (cf. Prinz, 1997).

Finally, we would like to emphasize that the present set of experiments has not been designed to decide between alternative accounts of location-specific PC effects. Whereas transfer of item-specific PC effects from manipulated to non-manipulated *items* may be a distinctive feature of control-based accounts and be inconsistent with learning-based accounts (cf. Braem et al., 2019), transfer of location-specific PC effects from manipulated to non-manipulated *locations*—or its absence—is consistent with both types of account, as we specify in more detail in the General Discussion.

## EXPERIMENT 1

Experiment 1, first, served to consolidate the findings of Hübner and Mishra (2016), who observed a Simon effect of 29 ms for mostly congruent locations and of 21 ms for mostly incongruent locations, hence a location-specific modulation of the Simon effect by only 8 ms. Second, we tested whether location-specific effects of conflict frequency in the Simon task would transfer from differently manipulated locations to equally distant non-manipulated locations, and if so, whether transfer regions would be the lateral or the vertical hemifields.

Transfer of conflict-adaptation effects from manipulated to non-manipulated locations requires sufficient PC effects for the manipulated locations in the first place. Therefore, transfer can only be expected for participants with regular PC effects for the manipulated locations. However, not all participants in a sample show even the most robust effects in experimental psychology, such as the Simon effect, but almost always some participants show null or even reverse effects. Therefore, we analyzed transfer not only for the complete sample, but also for the subsample of those participants who showed location-specific PC effects for the manipulated locations (i.e., numerically larger Simon effects for the mostly congruent location than for the mostly incongruent location). Due to noise in the data, this split is not exactly a separation of participants with and without location-specific PC effects, but there should be fewer participants without PC effects, and PC effects should be stronger, in the subsample than in the full sample. Transfer should thus be stronger as well (though statistical power is reduced because of the smaller number of degrees of freedom).

### Methods

#### Transparency and Openness Statement.

We report how we determined our sample size, all data exclusions (if any), all manipulations, and all measures in the study. The experiments reported here were not preregistered. The raw data from both experiments can be obtained from the first author upon request.

#### Participants.

We conducted a priori power analysis for determining the sample size of Experiment 1 with MorePower (Campbell & Thompson, 2012). Two previous studies provided useful effect sizes for this analysis. First, Hübner and Mishra (2016) reported an effect size of $\eta_{p}^{2}$ = .271 for the critical two-way interaction that reflected the location-specific proportion congruent effect in a Simon task. Second, Corballis and Gratton (2003, Experiment 3) reported an effect size of $\eta_{p}^{2}$ = .463 for the critical two-way interaction that reflected the transfer effect from manipulated (lateral) stimulus locations to the non-manipulated (central) stimulus location. We used the smaller effect size ($\eta_{p}^{2}$ = .27) for our power analysis, which revealed that a sample size of 38 participants would be required to detect an effect of this size with high power (1 − *β* = .95) and the alpha error set to .05. We decided to test 40 participants because this allowed us to assign 10 participants to each level of a technical factor (i.e., the locations of the high- and low-conflict conditions).

The sample consisted of 40 students with an average age of 22 years (range 19–25). The majority of the sample was female (*N* = 35), and right-handed (*N* = 35). All participants reported having normal or corrected-to-normal visual acuity and intact color vision. The participants gave written informed consent before the experiment began and received course-credits or payment (5 Euro). The sample was treated according to APA ethical standards^1^.

#### Apparatus and Stimuli.

The experiment was run in a dimly-lit laboratory at TU Dortmund University. Participants sat in front of a 19-inch color flat screen, with a viewing distance of approximately 60 cm. A computer program written with the software package EPrime 2.0 (Psychology Software Tools; Sharpsburg, PA, USA) controlled the timing of events in the experiment and recorded keypress responses and response times (RTs). Participants responded by pressing two keys (#4 and #6) on the number pad of a regular keyboard with the left and right index finger. The central axis of the number pad (i.e., the line through keys 2, 5, and 8) was aligned with the participant’s body midline.

**Figure 1.**
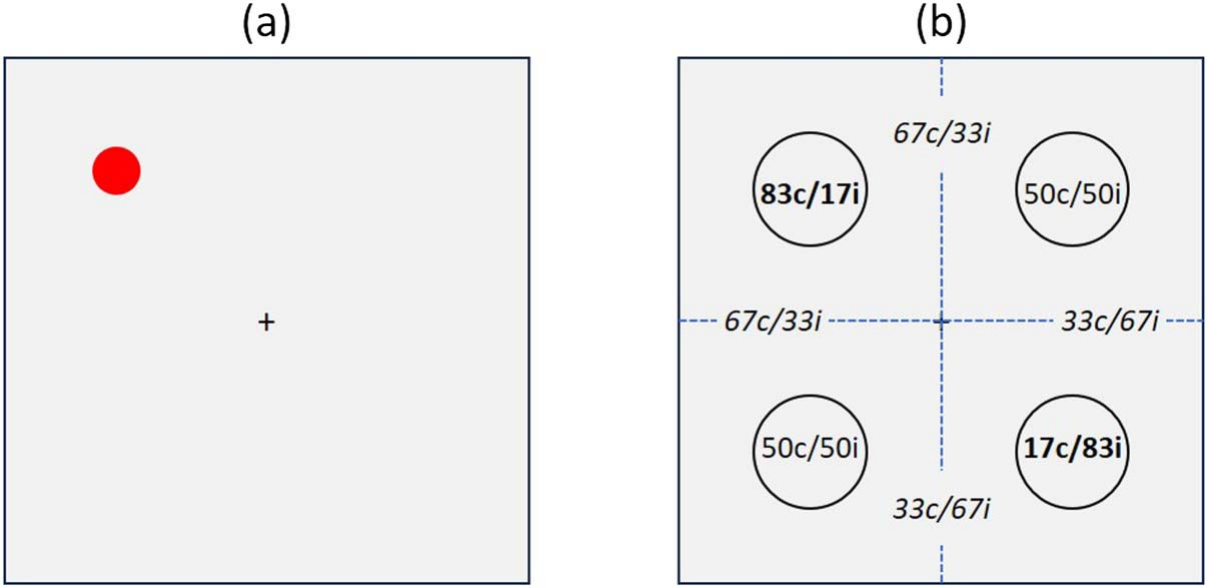
(A) Example of stimulus display as presented to the participant. (B) Percentages of congruent and incongruent stimuli at four peripheral stimulus locations are given in circles. Numbers in italics represent the overall percentages of congruent and incongruent stimuli in each lateral or vertical hemifield (c = congruent; i = incongruent).

A plus sign (4 × 4 mm) served as a fixation point at screen center. The imperative stimulus was a red or green circle with a diameter of 20 mm which could appear at one of five locations on the screen: four peripheral locations and one central location at the center of the screen (cf. Figure 1a). The peripheral locations (upper-left, upper-right, lower-right, and lower-left) were positioned at the corners of an imaginary square around screen center. The distance between the screen center and the stimulus center was 10 cm at each peripheral location, and thus the horizontal or vertical distance between two locations was 14.1 cm. All stimuli were shown on a light grey background.

#### Procedure.

Participants were seated at a desk with a keyboard and a computer screen. They received written instructions which explained the displays, a typical trial, and the participants’ task. In particular, instructions asked participants to fixate the fixation point and to press a left or right key to the color of a green or red circle that could appear at any of the five different locations. All participants in Experiment 1 were instructed to press the left key (#4) to the green stimulus and the right key (#6) to the red stimulus.

The experiment began with a practice block of 20 trials, which was followed by four test blocks of 128 trials each. Participants started each block by pressing a key on the keyboard. A trial began with a blank screen for one second, followed by the presentation of the fixation point. 500 ms later the color stimulus appeared at one of the five locations and remained on screen until the participant pressed a key or until two seconds had elapsed without a keypress. The fixation point remained at screen center when the colored stimulus appeared at a peripheral location, but was overwritten when the colored stimulus appeared at the central location. When participants had pressed the correct key within two seconds, the imperative stimulus was followed by a blank screen for one second. If participants had pressed the wrong key, or if participants had not responded within two seconds, an error message (in German) was shown for one second at screen center. Then the next trial began.

The most important manipulation concerned the frequency of red and green color stimuli at the five different locations in the four test blocks and thereby the relative frequency of congruent and incongruent stimuli at the different locations. In particular, we varied the relative frequency of congruent stimuli between two locations that were positioned on the same diagonal, while in the two remaining peripheral locations congruent and incongruent stimuli appeared with equal frequency. For example, for one quarter of the participants, mostly congruent stimuli (i.e., 87%) appeared in the upper-left location, mostly incongruent stimuli (i.e., 87%) in the lower-right location, and 50% congruent and incongruent stimuli in the remaining two locations (cf. Figure 1b).

If the mapping is green-left and red-right, then presenting mostly congruent stimuli at one peripheral location (e.g., green stimuli on the left side) and mostly incongruent stimuli at the other peripheral location (e.g., green stimuli on the right) implies that one stimulus color (green in this example) is presented more often than the other color (red in this example). In order to keep the overall frequency of the two colors, and hence the two responses, equal, we presented the color that was less frequent at the peripheral locations at the central location. This measure served to prevent effects of relative response frequency which could have increased, or even mimicked, the effects of conflict frequency.

In each test block, there were 24 (green and red) stimuli at each of the four peripheral locations and 32 stimuli (green or red) at the central location. We counterbalanced the locations of mostly congruent and mostly incongruent stimuli across participants. The frequencies of green and red stimuli at the five locations for each level of this technical factor are shown in Table 1.

**Table 1.** Absolute frequencies of stimulus colors at the five different stimulus locations used in the present experiments. The letters A–D represent four levels of a technical factor that was introduced to counterbalance the locations of mostly congruent and mostly incongruent stimuli across participants.

Level	Stimulus location				
	Upper-left	Upper-right	Lower-right	Lower-left	Center
A	20 green / 4 red	12 green / 12 red	20 green / 4 red	12 green / 12 red	32 red
B	4 green / 20 red	12 green / 12 red	4 green / 20 red	12 green / 12 red	32 green
C	12 green / 12 red	4 green / 20 red	12 green / 12 red	4 green / 20 red	32 green
D	12 green / 12 red	20 green / 4 red	12 green / 12 red	20 green / 4 red	32 red

#### Design and Data Analysis.

Experiment 1 rested on a three-factorial 2 × 2 × 2 design with *Location Type* (manipulated vs. non-manipulated), *Proportion* (mostly congruent vs. mostly incongruent), and *S-R Congruency* (congruent vs. incongruent) as within-participant factors. The factor *Location Type* codes whether the proportion of congruent trials was manipulated for a particular location or not. Hence, for participants faced with the conditions depicted in Figure 1b (or row A in Table 1), the upper-left and lower-right locations were manipulated locations, whereas the upper-right and lower-left locations were non-manipulated locations. The factor *Proportion* codes whether a particular location contained mostly congruent or mostly incongruent stimuli. This coding is clear for the manipulated diagonal. For the non-manipulated diagonal, we coded the location in the same lateral hemifield as the manipulated location with mostly congruent stimuli as mostly congruent, and the location in the same lateral hemifield as the manipulated location with mostly incongruent stimuli as mostly incongruent. That is, for participants with conditions depicted in Figure 1b (or row A in Table 1) the lower-left location was the mostly congruent location on the non-manipulated diagonal, whereas the upper-right location was the mostly incongruent location on the non-manipulated diagonal^2^. Finally, the factor *S-R Congruency* codes whether stimulus and response locations were congruent or incongruent with regard to the *horizontal* dimension. Dependent variables were RTs of trials with correct responses, and error percentages.

We analyzed the dependent variables, RT and accuracy, with three- and two-way ANOVAs as specified below. In addition to analyses involving the full sample, transfer effects to the non-manipulated locations were analyzed for the subsample of participants who showed regular location-specific PC effects at the manipulated locations (as defined by a positive difference between the Simon effect for the mostly congruent location minus the Simon effect for the mostly incongruent location).

For the RT analyses in both experiments, only RTs of correct responses were considered. In addition, we omitted the first trial of each block and trials with RT < 100 ms or RT > 1500 ms (less than 1% of the trials). For each participant and each of the 8 conditions defined by the combinations of the factors *Location Type, Proportion*, and *S-R Congruency* we computed the RT means and error percentages. We did not check for outliers in overall performance (e.g., overall RTs or overall error percentages of participants).

### Results

#### Response Times (RTs).

In the first step of our analysis RTs of correct responses were entered into a three-factorial ANOVA with *Location Type, Proportion*, and *S-R Congruency* as within-participant factors. Note that the transfer of location-specific effects of conflict frequency from manipulated to non-manipulated locations might lead to different patterns of results in this analysis, depending on the size and the transfer region. In particular, if transfer is strong to non-manipulated locations in the same lateral hemifields as the manipulated locations, the transfer effect will contribute to a strong Proportion × Congruency interaction, without a significant three-way interaction involving the factor Location Type, that is, without different PC effects for manipulated and non-manipulated locations. If, however, the PC effect is weaker for non-manipulated than for manipulated locations, the Proportion × Congruency interaction should come along with a significant three-way interaction. Finally, although not expected, it is possible that transfer would be to non-manipulated locations in the same vertical hemifield as the manipulated locations. In this case, with the present coding of the non-manipulated locations for the factor *Proportion*, a strong three-way interaction would be expected, with regular PC effects for the manipulated diagonal and inverted PC effects for the non-manipulated diagonal.

**Figure 2.**
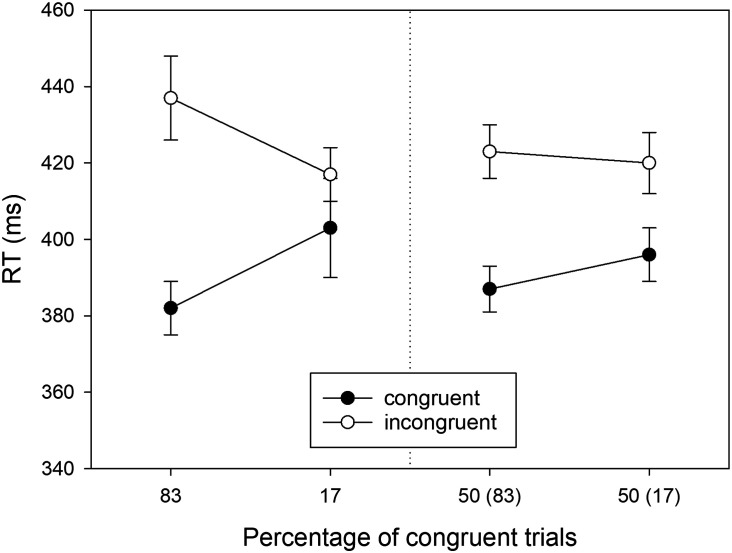
RTs observed in Experiment 1 as a function of Location Type (manipulated locations: left panel, non-manipulated locations: right panel), Percentage of congruent trials, and S-R Congruency (*N* = 40). For non-manipulated locations, the number in brackets gives the percentage of congruent trials for the manipulated location in the same horizontal hemifield. Error bars represent confidence intervals for within-subjects designs (Cousineau, 2017).

The observed means of the RTs in the eight conditions of our design are shown in Figure 2. Here we will only report the significant results; the results of the remaining *F* tests are reported in Appendix 1. Of three possible main effects, only the main effect of *S-R Congruency* was significant, *F*(1, 39) = 147.502, *MSE* = 559.027, *p* < .001, $\eta_{p}^{2}$ = .791. RTs were shorter in congruent conditions (*M* = 393 ms, *SD* = 52) than in incongruent conditions (*M* = 424 ms, *SD* = 50). Hence, there was an overall Simon effect of 31 ms. Of three possible two-way interactions, only the *Proportion* × *S-R Congruency* interaction was significant, *F*(1, 39) = 10.330, *MSE* = 1361.211, *p* = .003, $\eta_{p}^{2}$ = .209. This proportion-congruent effect reflected a larger Simon effect for mostly congruent locations (*M* = 45 ms, *SD* = 34) than for mostly incongruent locations (*M* = 19 ms, *SD* = 40). Finally, the three-way interaction was also significant, *F*(1, 39) = 13.506, *MSE* = 299.411, *p* < .001, $\eta_{p}^{2}$ = .257. According to the means of Figure 2, the transferred location-specific PC effect in the non-manipulated locations was smaller than the original effect in the manipulated locations or even unreliable or absent.

In the second step of our analysis we ran separate two-way ANOVAs (*Proportion* × *S-R Congruency*) for locations on the manipulated diagonal and locations on the non-manipulated diagonal. For the manipulated locations (cf. Figure 2, left), we observed a significant Simon effect (i.e., main effect of *S-R Congruency*) of 34 ms, *F*(1, 39) = 67.114, *MSE* = 701.394, *p* < .001, $\eta_{p}^{2}$ = .632. Moreover, there was a significant two-way interaction, reflecting a PC effect, *F*(1, 39) = 17.326, *MSE* = 958.127, *p* < .001, $\eta_{p}^{2}$ = .308. The Simon effect was larger for the mostly congruent (i.e., 83% congruent) location (*M* = 55 ms, *SD* = 37) than for the mostly incongruent (i.e., 17% congruent) location (*M* = 14 ms, *SD* = 44).

For the non-manipulated locations (cf. Figure 2, right), we observed a significant Simon effect (i.e., main effect of *S-R Congruency*) of 30 ms, *F*(1, 39) = 122.025, *MSE* = 293.148, *p* < .001, $\eta_{p}^{2}$ = .758. However, the two-way interaction of *Proportion × S-R Congruency* (i.e., the PC effect) failed to reach statistical significance, *F*(1, 39) = 2.153, *MSE* = 703.495, *p* = .150, $\eta_{p}^{2}$ = .052. Numerically, the Simon effect was larger for the location that shared the lateral hemifield with the mostly congruent location (*M* = 36 ms, *SD* = 29) than for the location that shared the lateral hemifield with the mostly incongruent location (*M* = 24 ms, *SD* = 34).

We complemented the analysis of mean reaction times by an analysis of the relation between the individual location-specific PC-effects for manipulated locations and the transferred location-specific PC-effects for non-manipulated locations. The correlation between these measures (which were differences of differences of RTs) was *r* = .65, *t*(38) = 5.23, *p* < .001.

#### Error Percentages.

**Figure 3.**
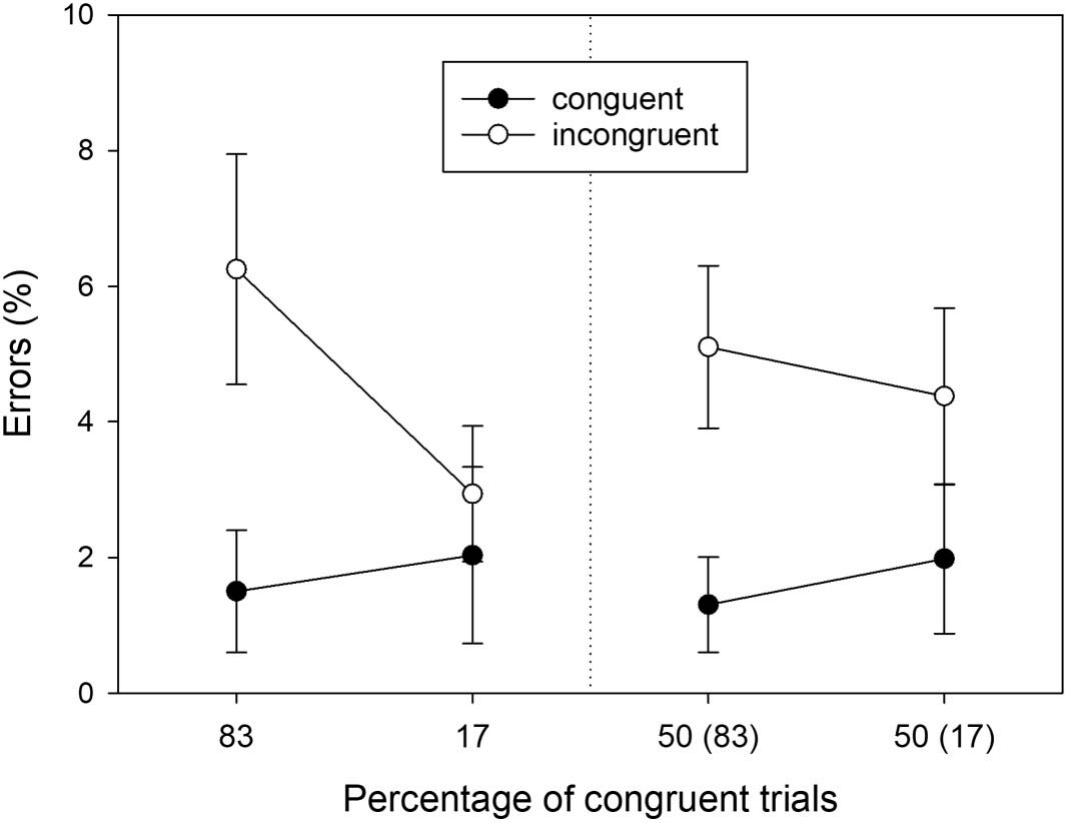
Error percentages observed in Experiment 1 as a function of Location Type (manipulated locations: left panel, non-manipulated locations: right panel), Percentage of congruent trials, and S-R Congruency (*N* = 40). For non-manipulated locations, the number in brackets gives the percentage of congruent trials for the manipulated location in the same horizontal hemifield. Error bars represent confidence intervals for within-subjects designs (Cousineau, 2017).

The means of the error percentages in the eight conditions of our design are shown in Figure 3. Error percentages were entered into a three-factorial ANOVA with *Location Type, Proportion*, and *S-R Congruency* as within-participant factors. We will only report the significant results here; the results of the remaining *F* tests are reported in Appendix 1. Of three possible main effects, only the main effect of *S-R Congruency* was significant, *F*(1, 39) = 21.808, *MSE* = 32.218, *p* < .001, $\eta_{p}^{2}$ = .359. Errors were less frequent in congruent conditions (*M* = 1.703, *SD* = 2.266) than in incongruent conditions (*M* = 4.667, *SD* = 6.102). Of three possible two-way interactions, only the *Proportion* × *S-R Congruency* interaction was significant, *F*(1, 39) = 8.427, *MSE* = 16.354, *p* = .006, $\eta_{p}^{2}$ = .178. This PC effect reflected a larger Simon effect in errors (*M* = 4.276, *SD* = 6.876) for mostly congruent locations as compared to the Simon effect in errors (*M* = 1.651, *SD* = 4.935) for mostly incongruent locations. Different from RTs, the three-way interaction was not statistically significant.

#### RT Analysis for the Reduced Sample.

**Figure 4.**
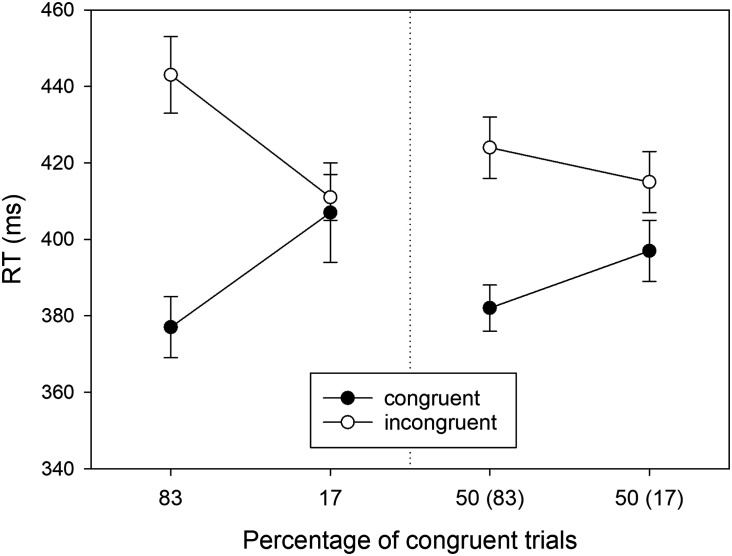
RTs observed for the reduced sample of Experiment 1 as a function of Location Type (manipulated locations: left panel, non-manipulated locations: right panel), Percentage of congruent trials, and S-R Congruency (*N* = 30). For non-manipulated locations, the number in brackets gives the percentage of congruent trials for the manipulated location in the same horizontal hemifield. Error bars represent confidence intervals for within-subjects designs (Cousineau, 2017).

For this analysis, we included a subgroup of 30 participants with positive location-specific PC effects (i.e., a larger Simon effect at the low-conflict location than at the high-conflict location), which ranged from 3 to 190 ms. Neglected were 10 participants who had negative location-specific PC effects (i.e., larger Simon effects at high-conflict location than at low-conflict location), which ranged from −1 to −65 ms. Taking the random variation of RTs—and location-specific PC effects derived from them—into account, this is not a strict selection of a subgroup with “true” positive PC effects, but it is an approximation. The mean RTs of this subgroup are shown in Figure 4.

The enhanced PC effect in this subgroup, as compared with the full sample (see Figure 4, left, as compared with Figure 2, left), is a trivial consequence of the selection. Therefore, we analyzed only the transfer data (Figure 4, right). There was a significant Simon effect (i.e., main effect of *S-R Congruency*) of 30 ms, *F*(1, 29) = 78.483, *MSE* = 349.444, *p* < .001, $\eta_{p}^{2}$ = .730. Crucially, the two-way interaction of *Proportion × S-R Congruency* (i.e., the PC effect) was significant, *F*(1, 29) = 5.689, *MSE* = 758.939, *p* = .024, $\eta_{p}^{2}$ = .164. The Simon effect (*M* = 42 ms, *SD* = 29) was larger for the non-manipulated location sharing the lateral hemifield with the mostly congruent location as compared to the Simon effect (*M* = 18 ms, *SD* = 37) for the non-manipulated location sharing the lateral hemifield with the mostly incongruent location.

### Discussion

Experiment 1 produced two main results. First, we observed location-specific effects of conflict frequency in a Simon task, and thereby replicated the only previous report of such effects (Hübner & Mishra, 2016). In our experiment, the Simon effect was larger at the mostly congruent location (55 ms) than at the mostly incongruent location (14 ms). Hence, the location-specific modulation of the Simon effect (for the complete sample) amounted to 41 ms in our experiment, which is much larger than the 8 ms modulation observed by Hübner and Mishra (2016). The differences in effect size might be related to several methodological differences between the studies. The most important ones are probably, first, the larger difference between high and low conflict frequencies (83% vs. 17%) in our experiments than in the study of Hübner and Mishra (2016; 75% vs. 25%) and, second, the larger distance between manipulated stimulus locations in our study (20 cm along the diagonal rather than 10.5 cm along the vertical). Both variables might foster the occurrence of location-specific PC effects (cf. Kuratomi & Yoshizaki, 2013, for similar arguments).

The second main finding of Experiment 1 is the transfer of location-specific effects of conflict frequency within lateral hemifields: we observed a larger Simon effect for the non-manipulated location that shared the lateral hemifield with the mostly congruent location than for the non-manipulated location that shared the lateral hemifield with the mostly incongruent location. The transfer within lateral hemifields, however, was statistically non-significant for the full sample. To disambiguate this statistical result, we re-ran the analysis for a subsample of 30 participants (from the overall sample of 40 participants) who had a positive (regular) location-specific effect of conflict frequency for the manipulated locations, and for this subsample the transfer was significant. In addition, for the full sample the transfer is also indicated by the significant correlation of 0.65 between the location-specific PC effects observed for the manipulated and the non-manipulated locations.

Notably the transfer within lateral hemifields was found even though each non-manipulated location was equally distant—or equally similar in terms of spatial codes—to both manipulated locations. Thus, the physical stimulus configuration was neutral with respect to transfer within lateral or vertical hemifields. The observed transfer of conflict-frequency effects is consistent with the hypothesis of separate mechanisms for conflict adaptation in the two cerebral hemispheres (Corballis & Gratton, 2003; Kuratomi & Yoshizaki, 2013).

## EXPERIMENT 2

In Experiment 2 we tested whether the transfer of conflict-frequency effects within lateral hemifields could be replicated when the response positions differed vertically rather than laterally as in Experiment 1. The spatial arrangement of stimulus locations remained the same but, depending on stimulus color, participants now had to press an upper or lower key. When stimuli and responses vary on the vertical dimension, a Simon effect for the vertical dimension can be expected (e.g., Ansorge & Wühr, 2004; Hedge & Marsh, 1975). According to the hypothesis of distinct mechanisms for conflict adaptation in the two cerebral hemispheres (Corballis & Gratton, 2003; Kuratomi & Yoshizaki, 2013), transfer should still occur within lateral visual hemifields even with vertically arranged responses. Alternatively, we envisaged the possibility that the response requirements affect the coding of (irrelevant) stimulus locations by emphasizing discrimination along the vertical, that is, along the spatial dimension that is relevant for response discrimination. In this case transfer would occur within the vertical rather than the lateral hemifields.

### Methods

#### Participants.

The sample for Experiment 2 consisted of 44 students with an average age of 22 years (range 19–30). The majority of the sample was female (*N* = 37), and right-handed (*N* = 39). All participants reported having normal or corrected-to-normal visual acuity and intact color vision. The participants gave written informed consent before the experiment began and were compensated by course-credits or payment (5 Euro).

#### Apparatus, Stimuli, and Procedure.

Apparatus, stimuli, and procedure were the same as in Experiment 1 except for response requirements and S-R mappings. Participants responded by pressing the “0” key at the bottom or the “/” key^3^ at the top of the number pad of a regular keyboard in response to the green or red stimulus, respectively. One half of the sample pressed the lower key with their left index finger and the upper key with their right index finger, the other half had the opposite finger-key assignment.

The frequency of red and green colored stimuli was manipulated as in Experiment 1, but S-R congruency now varied with regard to the vertical rather than the horizontal dimension. Again, we counterbalanced the locations of mostly congruent and mostly incongruent stimuli across participants, which resulted in four levels of this technical factor. The frequencies of green and red stimuli at the five locations for each level of the technical factor are shown in Table 1.

#### Design and Data Analysis.

Experiment 2 rested on a three-factorial 2 × 2 × 2 design with *Location Type* (manipulated vs. non-manipulated), *Proportion* (mostly congruent vs. mostly incongruent), and *S-R Congruency* (congruent vs. incongruent) as within-subject factors. The factor *Location Type* codes whether the proportion of congruent trials was manipulated for a particular location or not (cf. Figure 1, and Table 1). The factor *Proportion* codes whether a particular location contained mostly congruent or mostly incongruent stimuli. For the non-manipulated diagonal, we coded the location in the same lateral hemifield as the manipulated location with mostly congruent stimuli as mostly congruent, and the location in the same lateral hemifield as the manipulated location with mostly incongruent stimuli as mostly incongruent. Finally, the factor *S-R Congruency* codes whether stimulus and response locations were congruent or incongruent with regard to the *vertical* dimension. Dependent variables were response times (RTs) from trials with correct responses and error percentages. The statistical analyses were the same as for Experiment 1. We have also performed an exploratory analysis comparing the RT results from both experiments. The results of this analysis are reported in Appendix 2.

### Results

#### Response Times (RTs).

**Figure 5.**
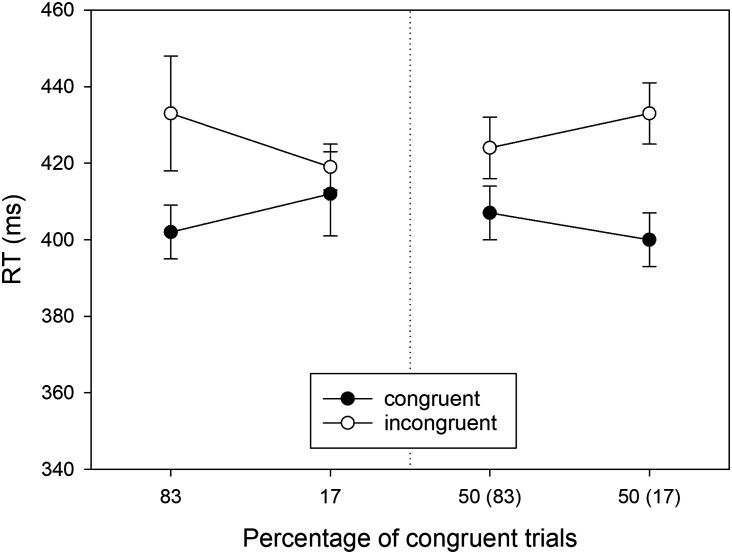
RTs observed in Experiment 2 as a function of Location Type (manipulated locations: left panel, non-manipulated locations: right panel), Percentage of congruent trials, and S-R Congruency (*N* = 44). For non-manipulated locations, the number in brackets gives the percentage of congruent trials for the manipulated location in the same horizontal hemifield. Error bars represent confidence intervals for within-subjects designs (Cousineau, 2017).

The mean RTs of correct responses in the eight conditions of our design are shown in Figure 5. In the first step of our analysis they were subjected to a three-factorial ANOVA with *Location Type, Proportion*, and *S-R Congruency* as within-participant factors. We will only report the significant results here; the results of the remaining *F* tests are reported in Appendix 1. From three possible main effects, only the main effect of *S-R Congruency* was significant, *F*(1, 43) = 80.809, *MSE* = 540.851, *p* < .001, $\eta_{p}^{2}$ = .653. RTs were shorter in congruent conditions (*M* = 405 ms, *SD* = 53) than in incongruent conditions (*M* = 428 ms, *SD* = 56). Hence, there was an overall Simon effect of 23 ms. None of the three possible two-way interactions was significant, all *F*(1, 43) < 1, but the three-way interaction was, *F*(1, 43) = 7.489, *MSE* = 1151.006, *p* = .009, $\eta_{p}^{2}$ = .148.

In the second step of our analysis we performed separate two-way ANOVAs (*Proportion* × *S-R Congruency*) for locations on the manipulated diagonal and locations on the non-manipulated diagonal. For the manipulated locations (cf. Figure 5, left), we observed a significant Simon effect (i.e., main effect of *S-R Congruency*) of 19 ms, *F*(1, 43) = 14.200, *MSE* = 1154.106, *p* < .001, $\eta_{p}^{2}$ = .248. Moreover, there was a significant two-way interaction, reflecting a PC effect, *F*(1, 43) = 7.009, *MSE* = 877.690, *p* = .011, $\eta_{p}^{2}$ = .140. The Simon effect was larger for mostly congruent (i.e., 83% congruent) locations (*M* = 31 ms, *SD* = 52) than for mostly incongruent (i.e., 17% congruent) locations (*M* = 7 ms, *SD* = 37) .

For the non-manipulated locations (cf. Figure 5, right), we observed a significant Simon effect (i.e., main effect of *S-R Congruency*) of 25 ms, *F*(1, 43) = 50.646, *MSE* = 554.863, *p* < .001, $\eta_{p}^{2}$ = .541. Crucially, the significant two-way interaction of *Proportion × S-R Congruency* reflected a PC effect, *F*(1, 43) = 4.471, *MSE* = 625.111, *p* = .040, $\eta_{p}^{2}$ = .094. However, the Simon effect was *smaller* for the non-manipulated location in the lateral hemifield containing the manipulated location with mostly congruent trials (*M* = 17 ms, *SD* = 35) than for the non-manipulated location in the lateral hemifield containing the manipulated location with mostly incongruent trials (*M* = 33 ms, *SD* = 34). In other words, for the non-manipulated locations the Simon effect was larger for the location in the *vertical* hemifield of the manipulated location with a large proportion of congruent trials than for the location in the *vertical* hemifield of the manipulated location with a small proportion of congruent trials. Thus, transfer of location-specific PC effects was within vertical rather than lateral hemifields.

To directly compare the size of PC effects for manipulated and non-manipulated locations, we recoded conflict frequency for non-manipulated locations in terms of vertical hemifields. The PC effect for manipulated locations (*M* = 24 ms, *SD* = 59) was numerically larger than the PC effect for non-manipulated locations (*M* = 16 ms, *SD* = 50), but the difference was not statistically significant, *t*(44) = 0.964, *p* = .340, *d* = 0.145 (two-tailed). As in Experiment 1, the correlation of *r* = .54 between the individual PC effects in manipulated and non-manipulated locations was significant, *t*(42) = 4.15, *p* < .001.

#### Error Percentages.

**Figure 6.**
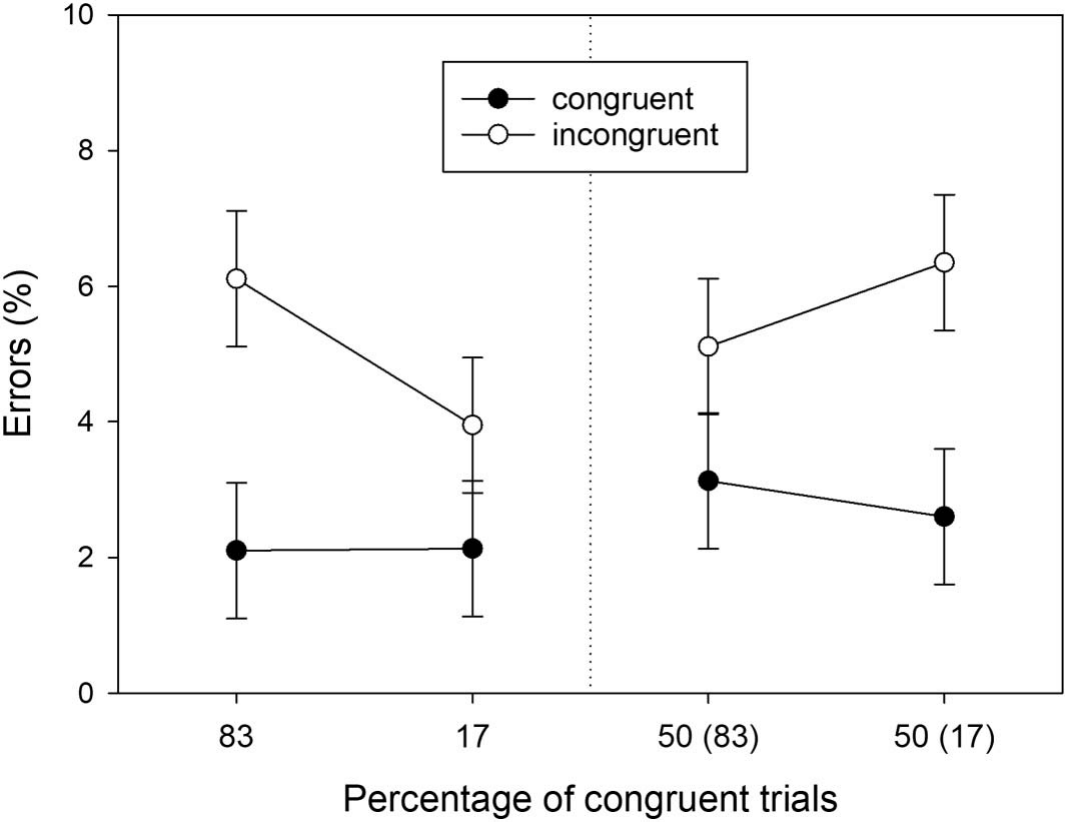
Error percentages observed in Experiment 2 as a function of Location (manipulated locations: left panel, non-manipulated locations: right panel), Percentage of congruent trials, and S-R Congruency (*N* = 44). For non-manipulated locations, the number in brackets gives the percentage of congruent trials for the manipulated location in the same horizontal hemifield. Error bars represent confidence intervals for within-subjects designs (Cousineau, 2017).

The means of the error percentages in the eight conditions of our design are shown in Figure 6. Error percentages were entered into a three-factorial ANOVA with *Location Type, Proportion*, and *S-R Congruency* as within-subject factors. We will only report the significant results here; the results of the remaining *F* tests are reported in Appendix 1. From three possible main effects, only the main effect of *S-R Congruency* was significant, *F*(1, 43) = 39.855, *MSE* = 18.419, *p* < .001, $\eta_{p}^{2}$ = .481. Errors were less frequent in congruent conditions (*M* = 2.491, *SD* = 3.583) than in incongruent conditions (*M* = 5.379, *SD* = 4.893). The only significant interaction was the three-way interaction, *F*(1, 43) = 6.120, *MSE* = 13.947, *p* = .017, $\eta_{p}^{2}$ = .125.

#### RT Analysis for Reduced Sample.

**Figure 7.**
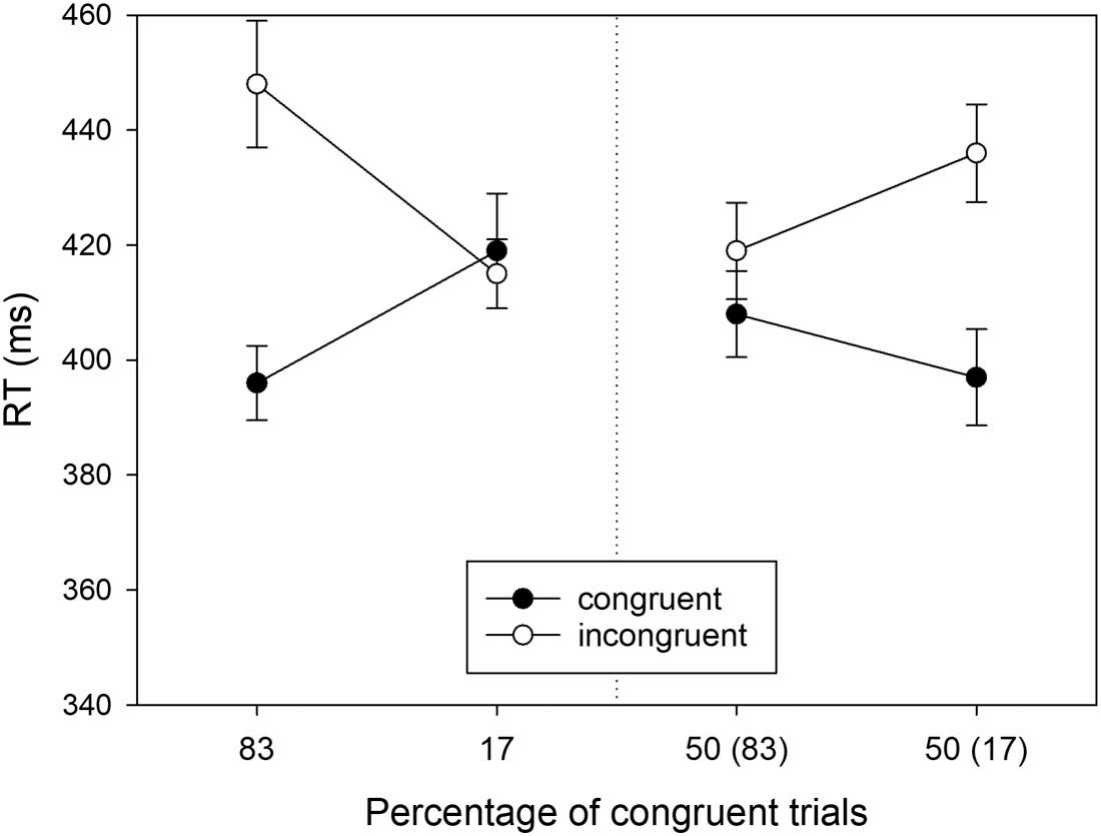
RTs observed for the reduced sample of Experiment 2 as a function of Location (manipulated locations: left panel, non-manipulated locations: right panel), Percentage of congruent trials, and S-R Congruency (*N* = 30). For non-manipulated locations, the number in brackets gives the percentage of congruent trials for the manipulated location in the same horizontal hemifield. Error bars represent confidence intervals for within-subjects designs (Cousineau, 2017).

For the sake of completeness, we also report the RT analysis for the subsample of participants (*N* = 30) who showed a positive location-specific PC effect for manipulated locations. The means are shown in Figure 7, and only the RTs for non-manipulated locations (Figure 7, right) were subjected to statistical analysis.

We observed a significant Simon effect (i.e., main effect of *S-R Congruency*) of 26 ms, *F*(1, 29) = 35.242, *MSE* = 554.242, *p* < .001, $\eta_{p}^{2}$ = .549. Crucially, the two-way interaction for non-manipulated locations, which reflects the transferred PC effect, was significant, *F*(1, 29) = 13.640, *MSE* = 402.562, *p* < .001, $\eta_{p}^{2}$ = .320. The Simon effect was *smaller* (*M* = 12 ms, *SD* = 31) for the non-manipulated location sharing the lateral hemifield with the mostly congruent location than for the non-manipulated location sharing the lateral hemifield with the mostly incongruent location (*M* = 39 ms, *SD* = 31). In other words, for the non-manipulated locations, the Simon effect was larger for the location in the vertical hemifield of the mostly congruent location than in the vertical hemifield of the mostly incongruent location—transfer of the location-specific PC effects occurred within vertical hemifields, and not within lateral hemifields as in Experiment 1.

### Discussion

Experiment 2 produced two main results. First, we observed location-specific effects of conflict frequency in a vertical Simon task, hence extending the results of Experiment 1 from the horizontal to the vertical Simon task. In particular, in Experiment 2, the vertical Simon effect was larger at the mostly congruent location (Simon effect = 31 ms) than at the mostly incongruent location (Simon effect = 8 ms). Hence, the location-specific modulation of the vertical Simon effect (for the complete sample) amounted to 23 ms in our experiment, which is numerically smaller than the modulation observed in Experiment 1 for the horizontal Simon effect (i.e., 41 ms)^4^.

The second main finding of Experiment 2 is the transfer of the location-specific PC effects to non-manipulated locations, but—importantly—this transfer now occurred within *vertical* hemifields. That is, in Experiment 2 we observed a larger Simon effect for the non-manipulated location that shared the vertical hemifield (but not the horizontal hemifield) with the mostly congruent location than for the non-manipulated location that shared the vertical hemifield with the mostly incongruent location. Together, the results of Experiments 1 and 2 reveal that response requirements affect the coding of (irrelevant) stimulus locations by biasing the coding towards the spatial dimension that is relevant for response discrimination.

## GENERAL DISCUSSION

In two experiments, we investigated the impact of varying the proportion of congruent stimuli between different stimulus locations on the size of the Simon effect, and the transfer of such location-specific PC effects from manipulated to non-manipulated locations. In the following we discuss our findings on both the location-specific effect of proportion congruent on the Simon effect and its transfer to other locations in turn.

### Location-Specific Effects of Conflict Frequency

The present study is the second one to demonstrate location-specific effects of conflict-frequency in the Simon task. In the only preceding study, Hübner and Mishra (2016) varied conflict frequency between vertical locations in a two-dimensional arrangement of stimulus locations, and investigated the location-specific PC effect on the horizontal Simon effect. They observed a location-specific PC effect of relatively small size. Our Experiment 1 replicates their finding with a numerically larger effect. The results of our Experiment 2 go beyond those of Hübner and Mishra (2016). Here, we showed location-specific PC effects for vertical Simon effects. The existence of location-specific PC effects along the lateral and the vertical dimension in the Simon task aligns with the results of Weidler et al. (2022) who reported similar effects for both dimensions with a variant of the flanker task. Thus, location-specific PC effects seem to be rather robust phenomena across different types of conflict tasks, suggesting similar mechanism of adjustment to conflict frequencies.

### The Possible Impact of Relative S-R Frequencies

Before we interpret the observed modulation of Simon effects at manipulated and non-manipulated locations as location-specific PC effects and their generalization, we need to refute an alternative account. According to this account, the observed modulation of Simon effects at different locations results from different relative S-R frequencies at the peripheral locations, which are not equalized by the centrally presented stimuli. In order to manipulate the proportion of congruent trials at two locations in opposite directions, one S-R pair had to be presented more frequently than the other S-R pair at both manipulated locations. For example, when the frequent congruent stimulus in the upper left location was green, the frequent incongruent stimulus in the lower right location was green as well. The different relative frequencies of S-R pairs at peripheral stimulus locations might have produced shorter latencies to peripherally more frequent S-R pairs (green in the example) than to peripherally less frequent S-R pairs. Such S-R frequency effect could, in principle, have mimicked the observed pattern of Simon effects. For example, a higher frequency of the left response to stimuli at peripheral locations in Experiment 1 could have sped up congruent responses to stimuli at left locations and thereby increased the Simon effect, but sped up incongruent responses to stimuli at right locations and thereby decreased the Simon effect. Similarly, a higher frequency of the upper response to stimuli at peripheral locations in Experiment 2 could have sped up the upper response, increasing the Simon effect for upper locations and decreasing the Simon effect for lower locations.

In order to avoid effects of relative S-R frequency in our experiments, we used a central location where we presented the peripherally less frequent S-R pair with a frequency that compensated for the difference at peripheral locations. Thus, globally—across all stimulus locations—relative S-R frequencies were identical. Hence, to account for the observed modulations of the Simon effect, the frequency-based account requires two assumptions. First, the effects of relative S-R frequency at peripheral stimulus locations should not be compensated by presenting the peripherally less frequent S-R pair at the central location. Second, provided that the first assumption is met, the effect of relative S-R frequency at one peripheral location should generalize to other peripheral locations where relative S-R frequencies are identical.

We tested these assumptions of a frequency-based account of the present findings in two separate experiments (Wühr & Heuer, 2025). In both experiments, there were four stimulus locations at the end points of an imaginary cross (i.e., left, right, upper, lower). In Experiment 1 we tested whether manipulating S-R frequency at the upper and lower locations would generalize to the non-manipulated left and right locations. Crucially, in contrast to the experiments reported here, there was no S-R congruency relation between upper/lower stimulus locations and left/right response locations. Hence, we investigated the impact of relative S-R frequency in isolation. Results showed that manipulating S-R frequency at the vertical locations not only affected performance at the vertical locations, but also at the non-manipulated horizontal locations. In other words, we observed a global effect of peripheral S-R frequency. This finding would be consistent with the second assumption of the frequency-based account if the first assumption would also hold.

The first assumption was tested in Experiment 2: does the presentation of the peripherally less frequent S-R pair at a central location compensate for the frequency difference at peripheral locations? The answer was affirmative. When the number of S-R presentations at the central location equaled the difference in the frequency of S-R pairs at peripheral locations, the frequency effect at manipulated locations was very small, namely 13 ms, as compared to 51 ms in Experiment 1 without the central stimuli. Thus, for the manipulated locations a small contribution of the different relative S-R frequencies to the observed modulation of the Simon effects seems possible, probably accounting for a part of the stronger modulation as compared with the one reported by Hübner and Mishra (2016). Crucially, with the central stimuli the transfer of relative-frequency effects from manipulated to non-manipulated locations was absent, actually slightly and non-significantly negative. Hence, the results of this control experiment reject the first assumption of a frequency-based account of the present results for the non-manipulated locations. We therefore conclude that differences in proportion congruent at manipulated locations are the main source of the modulation of Simon effects at the non-manipulated locations in the present experiments.

### Transfer Regions of Location-Specific Effects of Conflict Frequency

The two most important results of the present study are related to the transfer of location-specific PC effects from manipulated to non-manipulated stimulus locations. First, we observed transfer with a two-dimensional arrangement of stimulus locations, in which the two non-manipulated locations were equally distant from each manipulated location. Second, we observed that the region of transfer depended on the spatial arrangement of the response locations. With lateral arrangement of responses in Experiment 1 (left-right), the transfer between stimulus locations was within left or right lateral hemifields. In contrast, with vertical arrangement of responses in Experiment 2 (upper-lower), the transfer between stimulus locations was within lower or upper vertical hemifields. Thus, the boundaries between regions of transfer were related to the lateral or vertical separations of response locations.

Both results cannot be explained by existing accounts of the coding of stimulus locations as it shapes the transfer of location-specific PC effects. In most previous studies, conflict frequency varied between two lateral locations, situated in different visual hemifields (i.e., to the left and right of fixation), and a non-manipulated (transfer) location was located in the vicinity of each manipulated location (e.g., Pickel et al., 2019; Weidler & Bugg, 2016; Weidler et al., 2020, 2022). In these studies, transfer typically followed the spatial proximity between manipulated and non-manipulated locations. However, because in our experiments the two non-manipulated locations were equidistant from each manipulated location, (differences in) spatial proximity cannot explain the occurrence and direction of transfer. This does not preclude, however, that differences in spatial proximity cannot also affect the occurrence and direction of transfer in other situations.

Weidler and colleagues proposed an account of spatial-transfer effects in terms of categorical coding of stimulus locations (e.g., Weidler & Bugg, 2016; Weidler et al., 2020, 2022). According to this account, participants encode stimulus locations in terms of spatial categories (e.g., left vs. right; upper vs. lower; upper-left vs. lower-right). At manipulated locations, conflict frequency becomes associated to the categorical location code of this location. The associated conflict frequency can transfer from a manipulated location to a non-manipulated location that belongs to the same spatial category.

According to the categorical-coding account by Weidler and colleagues (e.g., Weidler & Bugg, 2016; Weidler et al., 2020, 2022), transfer effects would not be expected in our task in which each non-manipulated location was equally distant from both manipulated locations. With the two-dimensional arrangement, the four stimulus locations in our task could be classified into four spatial categories: upper-left, upper-right, lower-right, lower-left (cf. Weidler & Bugg, 2016). When the manipulated upper-left location is associated with frequent conflict, then this location shares the lateral code with the non-manipulated lower-left location and the vertical code with the non-manipulated upper-right location. Hence, according to the categorical-coding account, transfer is equally possible in both directions, and therefore we should not have observed differences in congruency effects between the two non-manipulated locations. The same conclusion holds under the assumption that transfer is governed by physical distances between manipulated and non-manipulated stimulus locations. In contrast to this prediction, we observed such differences, and the direction of these differences was affected by the spatial arrangement of the responses.

In contrast to the categorical-coding account, the notion of distinct mechanisms of conflict regulation in each cerebral hemisphere (Corballis & Gratton, 2003; Kuratomi & Yoshizaki, 2013) is consistent with the left and right transfer regions observed in Experiment 1, but not the lower and upper transfer regions of Experiment 2. According to this hypothesis, effects of conflict frequency on congruency effects should always transfer within lateral hemifields. The results of Experiment 2 add to the evidence against the notion of distinct mechanisms of conflict regulation in the two cerebral hemispheres, such as the finding that location-specific PC effects are possible for two locations sharing the same lateral visual hemifield (e.g., Weidler et al., 2022).

We envisage four assumptions to account for the effect of the spatial arrangement of the responses on the regions within which effects of conflict frequency transfer. Three of these assumptions are fairly established, the fourth one is new. First, in tasks with a two-dimensional arrangement of stimulus locations, participants code these locations on both dimensions, even when stimulus location is irrelevant for the task at hand. Stimulus locations can be coded both in egocentric and allocentric frames of reference (Umiltà & Liotti, 1987; Wang et al., 2016). The assumption that stimulus locations are coded with regard to both dimensions receives support, for example, from the simultaneous observation of horizontal and vertical Simon effects in tasks in which stimulus and response locations vary on both dimensions simultaneously (e.g., Ansorge & Wühr, 2004, Experiment 2). The second assumption is justified by the very observation of location-specific PC effects: participants associate each stimulus location with the frequency of processing conflict at this location (e.g., Crump et al., 2008; Weidler & Bugg, 2016), and these associations trigger adjustments such as conflict adaptation and/or contingency learning. Third, the location-specific responses to conflict-frequency transfer from manipulated locations to those non-manipulated locations that are sharing the relative location code (or a part of it) with the manipulated location.

To these more or less established assumptions we add the claim, based on the present findings, that the spatial dimension that is relevant for response discrimination (Experiment 1: lateral; Experiment 2: vertical) influences the coding of stimulus locations in two-dimensional space. In particular, the spatial dimension that is most (or more) relevant for response discrimination has more weight for the coding of stimulus location than a spatial dimension that is not (or less) relevant for response discrimination. We refer to this idea as “weighted two-dimensional location coding”. At present we envisage three different variants of this notion which have different implications for the observed transfer.

The first variant of the notion of weighted two-dimensional location coding is categorical and obeys an all-or-none principle. It simply holds that for the two dimensions of the four categorical codes upper-left, upper-right, lower-right, lower-left weighting is 0 or 1, that is, one of the two dimensions is neglected. Thus, the remaining categorical codes would be left and right or low and high, and location-specific PC effects should transfer to all locations within the regions denoted by these categorical codes, that is, essentially within one of the lateral or vertical hemifields. The obvious problem then is to account for the observation that the PC-effects at the non-manipulated locations were smaller than those at the manipulated locations (although in Experiment 2 this difference was not statistically significant).

The second variant of the notion of weighted two-dimensional location coding gives up the all-or-none-principle so that the weighting of the two dimensions in the categorical codes is graded (e.g., “strongly” left and “weakly” up). With this gradation the observation of smaller PC-effects at non-manipulated locations relative to manipulated locations in our experiments could be accounted for by the superposition of a strong transfer within, e.g., the left hemifield and a weak transfer, e.g., within the upper hemifield. However, spatially transferred location-specific PC effects tend to be smaller than the original PC effects even under conditions under which such superposition of opposing adjustments to high and low conflict frequencies is at least unlikely (e.g., Corballis & Gratton, 2003; Weidler et al., 2020).

**Figure 8.**
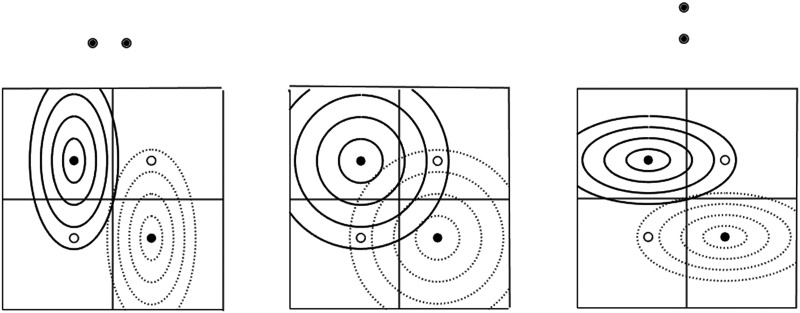
Illustration of weighted two-dimensional location coding with weights shaped by lateral (left) and vertical (right) response locations. Details explained in text.

The third variant, finally, is continuous and illustrated in Figure 8. Transfer is assumed to be a function of the “transfer distance” of a non-manipulated location from a manipulated one, where the transfer distance *δ* is a function of the weighted physical distances *d*_*x*_ and *d*_*y*_ on the two dimensions *x* and *y*: $\delta =\sqrt{{\left(w_{x}d_{x}\right)}^{2}+{\left(w_{y}d_{y}\right)}^{2}}$. In Figure 8 the manipulated locations are indicated by small filled circles and the non-manipulated locations by small open circles. Continuous and dotted circles or ellipses with increasing radii around the manipulated locations mark increasing transfer distances. In the central graph the isotropic case with equal weights *w*_*x*_ = *w*_*y*_ is illustrated. Here the transfer distances to the manipulated locations with opposite adjustments to conflict frequency are equal, so that the adjustments should superpose and thus cancel each other out. With lateral response locations (left graph of Figure 8), *w*_*x*_ > *w*_*y*_ (*w*_*x*_ = 2*w*_*y*_ in the graph) so that the lateral transfer distances are amplified for a given physical distance, whereas with vertical response locations (right graph of Figure 8), *w*_*x*_ < *w*_*y*_ (2*w*_*x*_ = *w*_*y*_ in the graph) so that the vertical transfer distances are amplified. With respect to transfer distances, response locations thus introduce anisotropy of transfer effects to non-manipulated locations in different directions.

### Implications for Accounts of Proportion-Congruent Effects

As described in the introduction, there are two classes of accounts for location-specific PC effects, control-based accounts and learning-based accounts. Control-based accounts assume that stimulus locations with a high proportion of incongruent items will more strongly trigger conflict-regulation processes than stimulus locations with a low proportion of incongruent items (e.g., Crump et al., 2006, 2017). In contrast, learning-based accounts assume that participants will learn location-specific associations between stimuli, locations, and responses that speed up responses to stimuli presented at mostly incongruent locations as compared to stimuli at mostly congruent locations (e.g., Schmidt, 2016).

The results of our experiments show that the effects of manipulating conflict frequency at one location (e.g., left-above) can generalize to a non-manipulated location when the two locations share spatial codes, and that response requirements can affect the direction of this transfer. Spatial transfer of PC manipulations seems compatible with transfer of location-specific control settings, and with transfer of learned location-specific S-R contingencies. A control-based account would simply have to assume that if attentional-control settings are established for a manipulated location, these control settings can transfer to another, non-manipulated, location that shares spatial codes with the manipulated location. Similarly, a learning-based account would have to assume that if a set of S-R associations has been learned for a manipulated location, this episodic representation can transfer to another, non-manipulated location. To be more precise, the learning-based account would have to assume that when a non-manipulated location shares spatial codes with a manipulated location, then processing the non-manipulated location can trigger the retrieval of S-R episodes that have originally been associated with the manipulated location. In sum, the results of the present experiments are compatible with both control-based and learning-based accounts of locations-specific PC effects.

### Some Directions for Future Research

The notion of weighted two-dimensional location coding suggests several questions and directions for future research. A first question directly arises from the present experiments: How does transfer look like when the horizontal and the vertical dimension are both relevant for response discrimination? This could be tested in an experiment where participants have to press an upper-left key to one stimulus color and a lower-right key to the other stimulus color (one might also use four response locations). When both dimensions are relevant for response discrimination, the transfer regions should be essentially those depicted in the central panel of Figure 8. That is, transfer should be equally strong in both directions, and we should not observe differences between horizontally and vertically matching test locations.

A second question concerns the role of response modality for biasing the direction of transfer. Are spatial responses necessary to produce spatial biases in the transfer of adjustments to location-specific conflict frequencies? One might tackle this issue by repeating the experiments reported here, but to use vocal instead of manual responses. In our view, the spatial biases in transfer effects should also be induced by vocal responses, as long as the task requires discriminating vocal responses with reference to a particular spatial dimension (such as “left” and “right”).

Turning from the role of responses to the role of stimuli, future research might address the question of whether (and which) stimulus factors affect the direction of transfer. Possible candidates are features of the stimulus arrangement that alter its perceptual organization. For example, consider a task with four stimulus locations (same as in present experiments) and two response locations (upper-left, lower-right) that require response discrimination on both dimensions. Conflict frequency is manipulated on two diagonally opposite locations as in the present experiments. Regions in the visual field could be defined by contours (cf., Weidler et al., 2020, Experiment 3), such as vertical or horizontal ellipses similar to those in Figure 8 which each include one manipulated and one non-manipulated location. Transfer regions could be shaped by these ellipses.

A more subtle way of manipulating the perceptual organization would be to enhance the relative salience of one spatial dimension with regard to the other. In one condition, the salience of the difference between horizontal stimulus locations is increased over the difference between vertical locations by making the horizontal distances between stimulus locations larger than the vertical distances (in terms of perceptual organization: creating two pairs of vertically arranged locations). In a second condition, the salience of the difference between vertical stimulus locations is increased relative to the horizontal locations (creating two pairs of laterally arranged locations). In this case, the more salient dimension in the stimulus arrangement might dominate the regions of transfer, lateral and vertical hemifields, respectively.

Finally, combining manipulations of response and stimulus configurations, one might ask how their respective influences are combined in shaping the pattern of transfer of location-specific PC effects. For example, can the influence of laterally separated response locations be compensated by the influence of vertically amplified stimulus distances? Do such opposing influences obey some kind of averaging rule or a winner-takes-it-all principle?

### Summary and Conclusions

In two experiments, we showed that location-specific adjustments to differences in conflict frequency are also possible in the Simon task. Moreover, we observed that location-specific adjustments to differences in conflict frequency can transfer from manipulated to non-manipulated locations even when each of the two non-manipulated locations was equidistant from both manipulated locations. In this situation, transfer was equally likely or possible in the horizontal and in the vertical direction. We observed that the direction of transfer depended on the spatial dimension that was relevant for discriminating alternative responses. When participants had to choose between a left or right response in Experiment 1, adjustments to conflict frequency transferred from a manipulated location to a non-manipulated location that shared the horizontal location code with the manipulated location. When, however, participants had do choose between an upper or lower response in Experiment 2, adjustments to conflict frequency transferred from a manipulated location to a non-manipulated location that shared the vertical location code with the manipulated location. This finding implies that a spatial dimension that is required for response discrimination has also more weight for the coding of stimulus locations, and the spatial coding of conflict frequencies, as compared to a spatial dimension that is not required for response discrimination. In short, response requirements can bias the spatial coding of conflict frequencies.

## ACKNOWLEDGMENTS

We are grateful to Melanie Richter and Sarah Behrendt for help in data collection.

## FUNDING INFORMATION

The authors have not received any funding for performing this research.

## AUTHOR CONTRIBUTIONS

P.W.: Conceptualization; Formal analysis; Methodology; Project administration; Resources; Software; Validation; Visualization; Writing – original draft; Writing – review & editing. H.H.: Formal analysis; Validation; Visualization; Writing – original draft; Writing – review & editing.

## DATA AVAILABILITY STATEMENT

The raw data from both experiments can be obtained from the first author upon request.

## Notes

^1^ We did not obtain ethical approval for the experiments reported here. However, we confirm that the experiments were conducted in accordance with the Declaration of Helsinki, the ethical guidelines of the “Deutsche Gesellschaft für Psychologie” (German Society for Psychology), and the ethical standards of APA.^2^ It would have also been possible to code the non-manipulated locations according to the manipulated location in the same vertical hemifield. When the upper-left location is mostly congruent, then the lower-left location is in the same lateral hemifield as the mostly congruent location, whereas the upper-right location is in the same vertical hemifield as the mostly congruent location. Based on the hypothesis of Corballis and Gratton (2003), which predicts transfer of location-specific conflict adaptation within the same lateral hemifield, we coded the non-manipulated locations with regard to the horizontal dimension.^3^ The slash represents the mathematical division sign on this particular keyboard.^4^ The results of a statistical comparison are reported in Appendix 2.

## APPENDIX 1

**Table A1.** Results from the three-factorial Location Type × Proportion × S-R Congruency ANOVA on RTs obtained in Experiment 1 (*N* = 40). The degrees of freedom for this analysis were 1 and 39.

Source of Variance	*F*	*MSE*	*p*	$\eta_{p}^{2}$
Location Type	1.820	373.576	.185	.045
Proportion	0.408	464.438	.527	.010
**S-R Congruency**	147.502	559.027	<.001	.791
Location Type × Proportion	0.127	539.188	.724	.003
Location Type × S-R Congruency	0.889	435.515	.351	.022
**Proportion × S-R Congruency**	10.330	1,362.211	.003	.209
**Location Type × Proportion × S-R Congruency**	13.506	299.411	<.001	.257

*Note*. Significant sources of variance are printed in **bold**.

**Table A2.** Results from the three-factorial Location Type × Proportion × S-R Congruency ANOVA on error percentages obtained in Experiment 1 (*N* = 40). The degrees of freedom for this analysis were 1 and 39.

Source of Variance	*F*	*MSE*	*p*	$\eta_{p}^{2}$
Location Type	0.001	11.273	.978	<.001
Proportion	2.487	16.140	.123	.060
**S-R Congruency**	21.808	32.218	<.001	.359
Location Type × Proportion	4.061	9.170	.051	.094
Location Type × S-R Congruency	0.157	9.364	.694	.004
**Proportion × S-R Congruency**	8.427	16.354	.006	.178
Location Type × Proportion × S-R Congruency	2.150	13.817	.151	.052

*Note*. Significant sources of variance are printed in **bold**.

**Table A3.** Results from the three-factorial Location Type × Proportion × S-R Congruency ANOVA on RTs obtained in Experiment 2 (*N* = 44). The degrees of freedom for this analysis were 1 and 43.

Source of Variance	*F*	*MSE*	*p*	$\eta_{p}^{2}$
Location Type	0.063	341.756	.802	.001
Proportion	0.017	414.494	.896	<.001
**S-R Congruency**	80.809	540.851	<.001	.653
Location Type × Proportion	0.479	460.774	.493	.011
Location Type × S-R Congruency	0.672	1,168.119	.417	.015
Proportion × S-R Congruency	0.929	351.795	.340	.021
**Location Type × Proportion × S-R Congruency**	7.489	1,151.006	.009	.148

*Note*. Significant sources of variance are printed in **bold**.

**Table A4.** Results from the three-factorial Location Type × Proportion × S-R Congruency ANOVA on error percentages obtained in Experiment 2 (*N* = 44). The degrees of freedom for this analysis were 1 and 43.

Source of Variance	*F*	*MSE*	*p*	$\eta_{p}^{2}$
Location Type	3.509	13.162	.068	.075
Proportion	0.824	13.471	.369	.019
**S-R Congruency**	39.855	18.419	<.001	.481
Location Type × Proportion	3.513	12.637	.068	.076
Location Type × S-R Congruency	0.002	22.288	.963	<.001
Proportion × S-R Congruency	0.098	10.639	.756	.002
**Location Type × Proportion × S-R Congruency**	6.120	13.947	.017	.125

*Note*. Significant sources of variance are printed in **bold**.

## APPENDIX 2

Here we report the results of an exploratory analysis comparing the RT results from Experiment 1 and Experiment 2. The major purpose of this analysis was to address three questions: First, regarding the manipulated locations, did congruency effects and/or proportion-congruent (PC) effects differ between the two experiments? Second, regarding the non-manipulated locations, was there significant transfer when looking at both experiments? Third, also regarding the non-manipulated locations, did the size of transfer differ between Experiment 1, with horizontally arranged responses, and Experiment 2, with vertically arranged responses?

We first analyzed RTs of correct responses from *manipulated* locations in a 2 (Experiment) × 2 (Proportion Congruent) × 2 (S-R Congruency) ANOVA, with Experiment as a between-subject factor, and Proportion Congruent and S-R Congruency as within-subject factors. The main effect of S-R Congruency was significant, *F*(1, 82) = 64.13, *MSE* = 938.79, *p* < .001, $\eta_{p}^{2}$ = .44, reflecting an overall Simon effect of 27 ms (*SD* = 31). The Simon effect was larger in Experiment 1 (*M* = 34 ms, *SD* = 27) than in Experiment 2 (*M* = 19, *SD* = 34), as indicated by a significant two-way interaction of S-R Congruency × Experiment, *F*(1, 82) = 5.03, *MSE* = 938.79, *p* = .028, $\eta_{p}^{2}$ = .06. The two-way interaction of Proportion Congruent × S-R Congruency was also significant, *F*(1, 82) = 23.71, *MSE* = 915.95, *p* < .001, $\eta_{p}^{2}$ = .22, reflecting a PC effect for manipulated locations across both experiments. The PC effect for manipulated locations (Experiment 1: 41 ms; Experiment 2: 24 ms) did not differ between the two experiments, as indicated by the nonsignificant three-way interaction, *F*(1, 82) = 1.67, *MSE* = 915.95, *p* = .200, $\eta_{p}^{2}$ = .02. All other F tests were not significant, all *F* < 1.00, all *p* > .480.

Next, we analyzed RTs of correct responses from non-manipulated locations in a 2 (Experiment) × 2 (Proportion Congruent) × 2 (S-R Congruency) ANOVA, with Experiment as a between-subject factor, and Proportion Congruent and S-R Congruency as within-subject factors. Note that, in this analysis, the factor Proportion Congruent codes whether a location was situated in the same horizontal hemifield as the manipulated location with mostly (i.e., 67%) congruent stimuli, or situated in the same horizontal hemifield as the manipulated location with mostly incongruent (i.e., 33% congruent) stimuli. The main effect of S-R Congruency was significant, *F*(1, 82) = 148.21, *MSE* = 430.39, *p* < .001, $\eta_{p}^{2}$ = .64, reflecting an overall Simon effect of 28 ms (*SD* = 21). The nonsignificant two-way interaction of S-R Congruency × Experiment, *F*(1, 82) = 1.04, *MSE* = 430.39, *p* = .310, $\eta_{p}^{2}$ = .01, indicates that Simon effects at non-manipulated locations did not differ between the two experiments. Moreover, the two-way interaction of Proportion Congruent × S-R Congruency was not significant, *F*(1, 82) = 0.10, *MSE* = 662.39, *p* < .747, $\eta_{p}^{2}$ < .01, reflecting the absence of a PC effect for non-manipulated locations across the experiments. However, this nonsignificant two-way interaction must be considered in combination with the significant three-way interaction, *F*(1, 82) = 6.31, *MSE* = 662.39, *p* = .014, $\eta_{p}^{2}$ = .07. Notably the significant three-way interaction reflects opposite patterns of PC effects at non-manipulated locations for Experiment 1 and Experiment 2: Whereas the Simon effect in Experiment 1 was larger for the nonmanipulated location in the lateral hemifield with mostly congruent stimuli relative to the nonmanipulated location in the lateral hemifield with mostly incongruent stimuli, the opposite pattern emerged in Experiment 2. In other words, the pattern in Experiment 2 reflected the finding of a larger Simon effect for the nonmanipulated location in the vertical hemifield with mostly congruent stimuli relative to the nonmanipulated location in the vertical hemifield with mostly incongruent stimuli. All other *F* tests were not significant, all *F* < 1.20, all *p* > .250.

Finally, we directly compared the size of the location-specific PC effects at the non-manipulated locations between Experiments 1 and 2. Therefore, we first recoded the location-specific PC effects for the non-manipulated location in Experiment 2 in such a way that the proportion of congruent items varied between vertical hemifields, consistent with the response locations. The location-specific PC effects for non-manipulated locations amounted to 12 ms (*SD* = 53) for Experiment 1 and 16 ms (*SD* = 50) for Experiment 2, with the difference being non-significant, *t*(82) = 0.32, *p* = .747, *d* = 0.07.
